# Supplementary material for: A Rationale for the Activity of Bone Target Therapy and Tyrosine Kinase Inhibitor Combination in Giant Cell Tumor of Bone and Desmoplastic Fibroma: Translational Evidences
Source: Biomedicines. 2022 Feb 3;10(2):372. doi: 10.3390/biomedicines10020372 (PMC8962296; doi:10.3390/biomedicines10020372)
Supplement: Supplementary file 1 [file biomedicines-10-00372-s001.zip › biomedicines-1552264-supplementary.pdf]

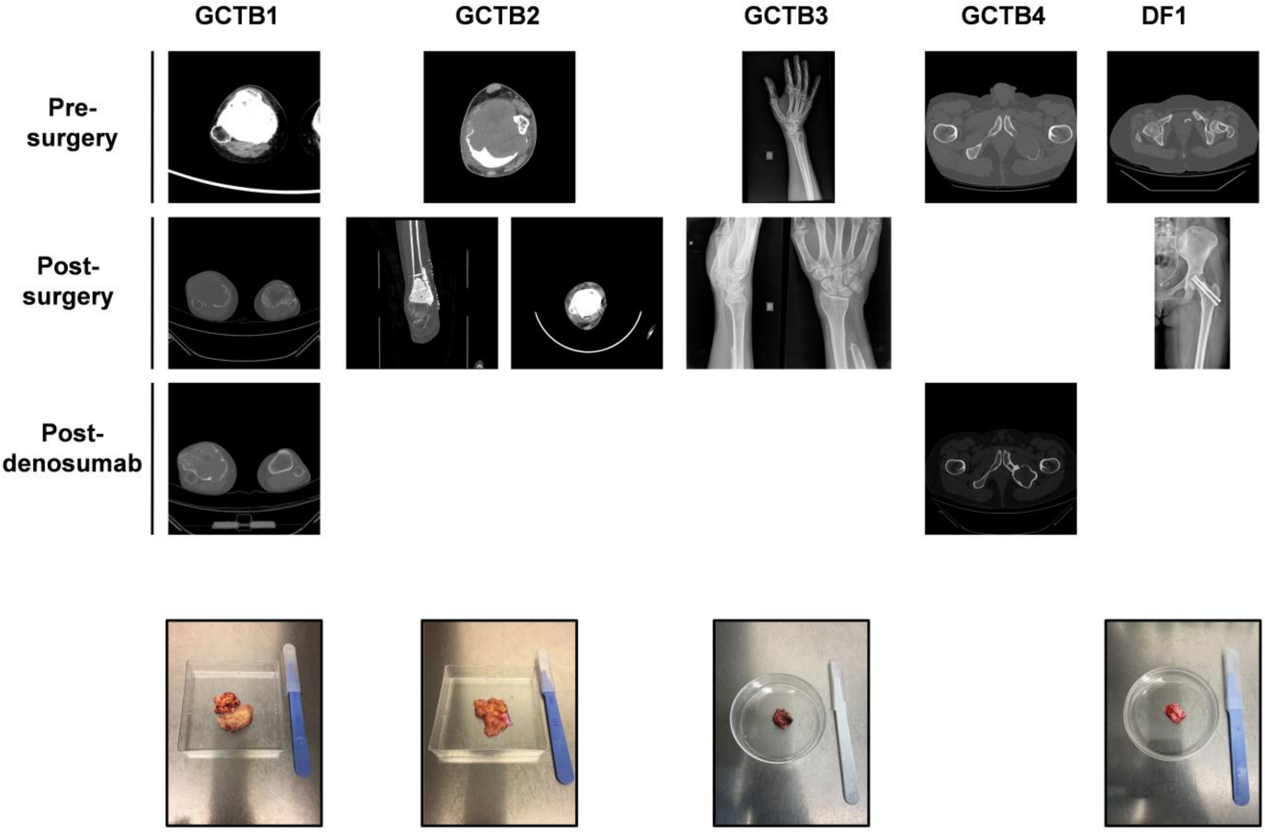

Figure S1. Representative X-ray or CT scan images of GCTB1, GCTB2, GCTB3, GCTB4, and DF1 patients pre-surgery, post surgery and post-denosumab treatment. In the lower panels, explanted tumor specimens used for patient-derived primary culture isolation.

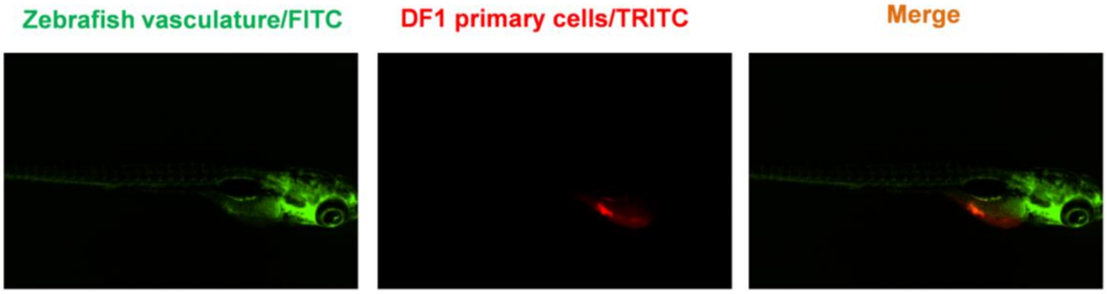

Figure S2. DF1 xenotransplanted zebrafish embryo in perivitelline duct representative images with A1r plus confocal microscopy at 72 hpi. Green probe stain zebrafish vasculature, red probe stain DF1 primary cells and images merged.

Table S1. Table showing relative expression of investigated target genes in GTCB1, GTB2, GTCB3 and DF1, with related p values. FC, Fold Change. Ct, threshold cycle (average of three replicates). In bold, FC with significant p value (<0.05). B2M was used as reference gene. B2M Cts average were: 21.3 for GCTB1, 21.5 for GCTB2, 21.04 for GCTB3, 23.1 for DF1. Healthy related tissues Cts were ≤ 35 for all genes analyzed.

Target genes	GCTB1			GCTB2			GCTB3			DF1		
	Ct	FC	p value	Ct	FC	p value	Ct	FC	p value	Ct	FC	p value
RANK	30.09	**8.72**	0.037	30.09	**21.33**	0.019	30.55	**10.19**	0.035	35.33	1.94	0.269
RANK-L	28.27	**92.51**	0.004	27.13	**234.33**	0.002	27.96	**92.54**	0.003	29.01	**59.82**	0.005
OPG	33.02	**0.35**	0.025	30.91	1.15	0.322	33.55	**0.15**	0.020	34.16	0.85	0.289
CXCR4	29.91	**25.43**	0.0003	30.16	**21.89**	0.001	32.14	**5.27**	0.029	31.71	**94.24**	0.0002
CXCL12	28.93	**0.06**	0.003	28.94	**0.07**	0.003	29.60	**0.03**	0.005	31.05	**0.25**	0.0009
OPN	27.77	**82.12**	0.0004	25.97	**329.26**	0.00002	25.54	**312.03**	0.00009	30.81	**44.13**	0.0001
RUNX2	27.59	**71.53**	0.049	26.98	**125.69**	0.039	28.30	**35.24**	0.034	30.24	**49.62**	0.029
FLT1	34.78	0.73	0.182	33.47	**2.75**	0.019	33.96	1.09	0.269	36.71	1.01	0.941

Table S2. Toxicology profile of DF1 xenotransplanted zebrafish embryos after exposure to DENO, LENVA and DENO + LENVA or untreated.

Conditions	Injected embryos 2 hpi	Alive embryos 72 hpi	Delta % 72 hpi vs 2 hpi
CTR	10	9	10%
DENO	8	6	25%
LENVA	9	5	44%
DENO + LENVA	9	0	100 %
